# Development of a Prognostic Model for Ovarian Cancer Patients Based on Novel Immune Microenvironment Related Genes

**DOI:** 10.3389/fonc.2021.647273

**Published:** 2021-03-31

**Authors:** Wei Wang, Qianqian Wu, Ziheng Wang, Shiqi Ren, Hanyu Shen, Wenyu Shi, Yunzhao Xu

**Affiliations:** ^1^ Department of Clinical Biobank, Nantong University Affiliated Hospital, Nantong, China; ^2^ Department of Medicine, Nantong University Xinling College, Nantong, China; ^3^ Department of Oncology, Affiliated Hospital of Nantong University, Nantong, China; ^4^ Department of Obstetrics and Gynecology, Nantong University Affiliated Hospital, Nantong, China

**Keywords:** immune microenvironment, ovarian cancer, biomarker, prognostic model, bioinformatics

## Abstract

Ovarian cancer (OV) has become the most lethal gynecological cancer. However, its treatment methods and staging system are far from ideal. In the present study, taking the advantage of large-scale public cohorts, we extracted a list of immune-related prognostic genes that differentially expressed in tumor and normal ovarian tissues. Importantly, an individualized immune-related gene based prognostic model (IPM) for OV patients were developed. Furthermore, we validated our IPM in Gene Expression Omnibus (GEO) repository and compared the immune landscape and pathways between high-risk and low-risk groups. The results of our study can serve as an important model to identify the immune subset of patients and has potential for use in immune therapeutic selection and patient management.

## Introduction

With patients often diagnosing at an advanced stage, Ovarian Cancer (OV) has become the most lethal gynecological cancer (1). Patients with OV may have no symptoms or mild symptoms until the cancer is in its advanced stages (1), which then responds poorly to treatment. According to the International Federation of Gynecology and Obstetrics (FIGO) staging system, treatments for OV patients usually include debulking surgery and adjuvant or neoadjuvant chemotherapy. However, even if patients have similar clinical characteristics and the same stage, clinical outcome of them may vary (2), so FIGO staging system currently used is far from ideal. As a result of the molecular heterogeneity, a large amount of OV patients develop metastases and relapses earlier than other patients. Gene expression of biomarkers in tumor tissues has been proved to be reliably related to clinical outcome (3, 4). Hence, in the context of additional clinical therapy, it is vital to identify the subcategory of patients with poor survival outcomes and higher mortality. In ovarian cancer, it is of primary importance to recognize a more comprehensive prognostic signature that includes the biological context. To do so, extensive databases of the biological characteristics and accessibility of all-encompassing public cohorts with data on their gene expression have been established.

Current first-line treatments for OV involve debulking surgery followed by chemotherapy and target therapy. Even though this initial therapy shows a better curative effect to more than 80% of patients, chemotherapy resistance will appear when most patients relapse (5). Also, the emergence of immunotherapy for the treatment of ovarian cancer has led to the rise of the most promising methodologies and options. The association of the abundance of tumor infiltrating cells (TIICs) with higher levels of survival for OV patients (6–8), remains evident. In light of this, immunotherapies possess crucial importance in enhancing cancer outcomes, which are also applicable to OV. Algorithms (9, 10) have been designed to predict the infiltration of TIICs. For instance, an algorithm named ESTIMATE (Estimation of STromal and Immune cells in MAlignant Tumor tissues using Expression data) (9), which develop by Yoshihara et al, could predict the infiltration of immune cells and stromal cells by calculating immune scores and stromal scores based on gene expression data from TCGA database. Many previous study have applied the algorithm to various types of cancer, such as breast cancer (11), colon cancer (12), gastric cancer (13) and brain cancer (14). Thus, the effectiveness of such big-data based algorithms has already been shown, although the utility on OV has not been studied in detail.

In the present study, taking the advantage of large-scale public cohorts, we extracted a list of immune-related prognostic genes that differentially expressed in tumor and normal ovarian tissues. Importantly, an individualized immune-related gene based prognostic model for OV patients was developed. Furthermore, we validated our immune prognostic model (IPM) in Gene Expression Omnibus (GEO) repository and compared the immune landscape and pathways between high-risk and low-risk groups. The results of our study can serve as an important model to identify the immune subset of patients and has potential for use in immune therapeutic selection and patient management.

## Methods and Materials

### Data Acquisition

388 gene expression profiling and the corresponding clinical information were downloaded from the Cancer Genome Atlas (TCGA) data portal (https://tcga-data.nci.nih.gov/tcga/) (up to July 10, 2019) (15). The gene expression profile matrix files and clinical information from GSE9891 based on platform Affymetrix Human Genome U133 Plus 2.0 Array (containing 285 OV samples) were obtained from Gene Expression Omnibus (GEO) repository (16). In addition, we downloaded the gene expression profiling of 88 normal ovarian tissues from the Genotype-Tissue Expression (GTEx) project, which used for comparing with tumor tissues (17). The next processing excluded cases lacking important clinical feature, such as age, stage, and overall survival. Finally, 374 samples from TCGA-OV and 275 samples from GSE9891 were retained for further study. Upon the discovery of data duplication, we utilized the average value of the RNA expression. We generated the data according to the policies of GEO and TCGA on their data accessibility and based our analyses on existing regulations and protocols.

### Recognition of Differentially Expressed Genes (DEGs)

We performed differentially expression analysis between high and low immune score groups (|*log*_2_ *FC|* > 1.8 and FDR<0.05), tumor and normal tissues (|*log*_2_ *FC|* > 1 and FDR<0.05). Package limma (18) was used to perform differentially expression analysis.

### Functional Enrichment Analysis

To analyze the DEGs we identified, we utilized the v 6.8 of the Database for Annotation, Visualization and Integrated Discovery (DAVID). Moreover, we also used an enrichment analysis known as gene ontology (GO) to verify the cellular components, signaling pathways, biological processes, and molecular functions that are linked to these differentially expressed genes (19). Our statistical significance level was set at a p-value of <0.05.

### Evaluation of Immune Infiltration Level

CIBERSORT algorithm was used to evaluate the proportion of tumor-infiltrating lymphocytes. CIBERSORT (20) is a widely accepted computing method to analyze immunological characteristics based on a gene expression signature matrix containing hundreds of marker genes. We downloaded a gene signature matrix with interpretation, known as the LM22, from the webpage of CIBERSORT (http://cibersort.stanford.edu/), which outlined 22 subtypes of immune cells. These cells are comprised of activated and resting dendritic cells, activated and resting NK cells, and activated and resting mast cells. Additionally, the 22 subtypes also include neutrophils and eosinophils, naive and memory B cells, plasma cells, seven kinds of T cells, M0-M2 macrophages, and monocytes. For every sample file, we accounted for the root mean squared error and p-value of the CIBERSORT to enhance the deconvolution algorithm’s accuracy. The number of permutations that the algorithm utilized under standard signature matrix was 100. For the succeeding analysis, we specifically chose and filtered data that had a CIBERSORT p-value<0.05. Additionally, using the algorithm of the CIBERSORT, we analyzed the immune cell fractions of the samples we generated from GEO and TCGA cohorts. The gene expression data across 32 different kinds of cancer from TCGA is comprised of 10,897 samples. TIMER conducts another set of analysis to compute the abundance of immune cells that have infiltrated the tumor. There are six subcategories and they are the neutrophils, CD8 T and CD4 cells, macrophages, dendritic cells, and B cells. This could be certainly used for discovering the association of TIICs with the other parameters. Then, we obtained the matrix of immune cells that are infiltrating the tumor of ovarian cancer patients. Finally, we computed the association of the IPM risk score with the immune infiltration.

### Association Between TFs and Immune-Related Prognostic Genes

To reveal potential regulatory mechanisms of immune-related prognostic genes, we explored which TFs have ability in regulating these genes. Cistrome Cancer (21) is a comprehensive web database that incorporates the TCGA’s data on cancer genomics that have accessible profiles of 23,000 chromatin and ChIP-seq, necessary to generate the governing association of TFs with the transcriptomes. Additionally, this is a valued system for empirical and computational study on the biology of cancer. Moreover, it encompasses 318 clinically relevant TFs that are responsible for establishing the governing network of the possible TFs and existing IRGs.

### Construction of Immune Prognostic Model

528 genes both differentially expressed in normal vs tumor tissues and high immune score vs. low immune score groups were analyzed *via* univariate Cox analysis to define the prognostic value of these genes. At P<0.05, we regarded the genes as statistically significant in this research. Original proportional hazards regression does not apply to genes that are highly associated, leading to our use of the least absolute shrinkage and selection operator (LASSO) with L1-penalty. This is a renowned methodology that evaluates the rules that predict and handle the collinearity (22). Using the LASSO method, we selected the primary immune genes from the significant cohort in the univariate analysis of the Cox regression. This approach enabled us to determine a sub-category of the immune genes that are included in the prognosis of hepatocellular carcinoma (HCC) patients. This was executed by taking into account the decrease of the regression coefficient through the pressing of a penalty comparative to their size. Finally, a small number of indicators that have a nonzero weight persisted while the majority of the possible indicators were contracted to zero. Hence, we applied the proportional hazards regression that has been calculated by LASSO to further decrease the presence of immune genes. In this study, we generated samples of an already existing sample dataset repeatedly for 1000 times. Then, we selected the immune genes that were repeated N900 times (23). Using the “glmnet” R package, we completed the LASSO Cox analysis (Version: 2.0–16; https://cran.r-project.org/web/packages/glmnet/index.html). Then, we established a prognostic immune-related model by using the beta coefficients from the multivariate Cox regression analysis. These coefficients are multiplied to the expression level of each immune gene. Finally, to investigate the best threshold or cutoff for patients with HCC, we applied the X-tile 3.6.1 software (Yale University, New Haven, CT, USA).

### Evaluation of Performance of Our IPM

Kaplan-Meier survival analysis and receiver operating characteristic (ROC) analyses were used to evaluate the accuracy of our IPM. To verify if the forecast of the prognostic model is independent of the conventional clinical features, multivariate Cox analysis after univariate selection were performed based on TCGA-OV dataset and GSE9891 dataset. Only samples with entire clinical information were subjected in this study.

## Results

### Immune Scores Are Significantly Associated With Immune Infiltration in OV

**Figure 1:**
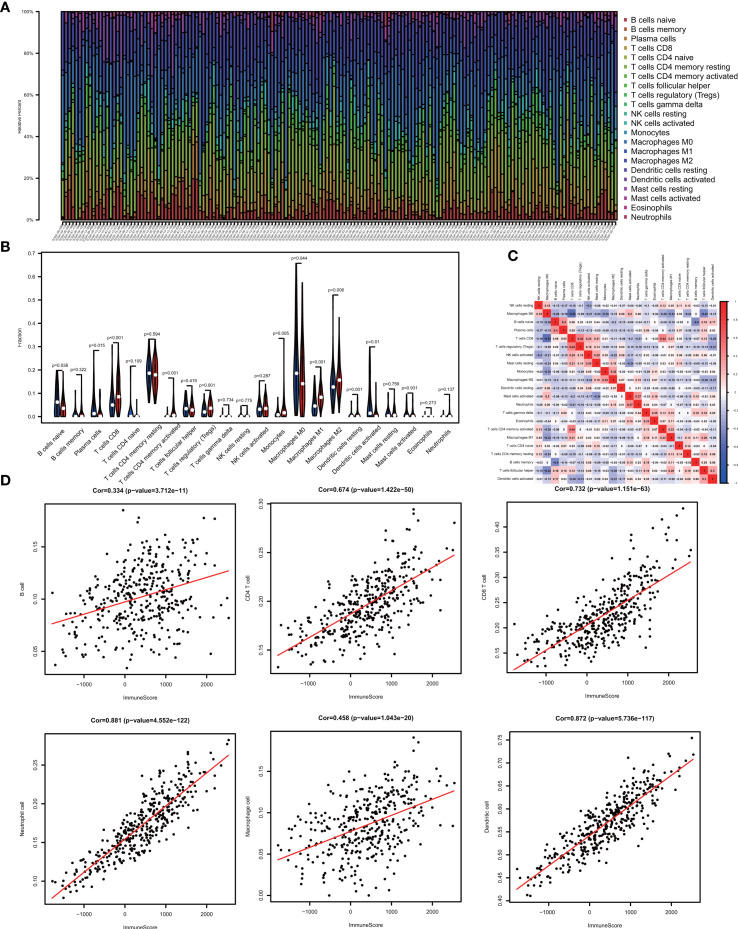
The landscape of immune infiltration in low and high immune score OV patients. **(A)** Relative fractions of immune cells in high and low immune score groups. **(B)** The proportion of different types of immune cells in high and low immune score groups. High immune score groups contains a higher proportion of T cells CD(P value=0.000), T cells CD4 memory activated (P value=0.000), T cells regulatory (Tregs) (P value=0.001), Monocytes (P value=0.005), Macrophages M1(P value=0.000), Macrophages M2 (P value=0.006) and Dendritic cells resting (P value=0.001), while B cells naïve (P value=0.038), Plasma cells (P value=0.015), Macrophages M0 (P value=0.044) and Dendritic cells activated (P value=0.001) were lower. **(C)** Correlation matrix of all 22 immune cell proportions. **(D)** Correlations between immune score and immune infiltrating level of 6 types of immune cells.

One of the reassuring approaches for the treatment of OV has been immunotherapy. To raise the efficacy of anti-tumoral immunotherapy, a fundamental factor must be taken into account is the microenvironment of the tumor. This is due to its composition that is largely comprised of immunosuppressive cell types that promote immune escape that also weaken the antitumor immunity (9, 24, 25). Numerous studies (12, 26, 27) have confirmed an immune score generated by the ESTIMATE algorithm was a reliable index to evaluate the concentration of the TIICs. As the TCGA database provided our access to the clinical characteristics and gene expression profiles of 388 ovarian cancer patients, we could confirm the performance of immune score in OV. The distribution of our immune scores occurred from -1,781.66 to 2529.21 based on the ESTIMATE algorithm. Then, we determined the ability of the immune score to forecast the infiltration of immune cells in OV by utilizing the recently reported CIBERSORT, which could evaluate the fraction of 22 kinds of TIICs. **Figure 1A** summarizes the outcome achieved from 388 OV patients. As is shown in **Figure 1B**, fractions of immune cells varied significantly among high and low immune score groups (based on the median value 411.13). Compared with low immune score groups, high immune score groups contains a higher proportion of T cells CD8 (P value=0.000), T cells CD4 memory activated (P value=0.000), T cells regulatory (Tregs) (P value=0.001), Monocytes (P value=0.005), Macrophages M1(P value=0.000), Macrophages M2 (P value=0.006) and Dendritic cells resting (P value=0.001), while B cells naïve (P value=0.038), Plasma cells (P value=0.015), Macrophages M0 (P value=0.044) and Dendritic cells activated (P value=0.001) were lower (**Table 1**). The quantity of immune cells differs across the groups. Hence, the proportional differences amongst the TIICs may serve as a representation of an essential immune characteristic that sets the two groups apart. Additionally, the weak to moderate correlation of the various subpopulations of tumor-infiltrating lymphocytes were evident (**Figure 1C**). Correlations were analyzed between the immune scores and the immune cell infiltration level based on the table matrix (the density of 6 types of immune cells in all TCGA samples) downloaded from TIMER (https://cistrome.shinyapps.io/timer/). Immune scores showed a moderate to strong correlation with tumor-infiltrating immune cells (**Figure 1D**). Above all, immune score developed by Yoshihara et al. is a reliable indicator of immune infiltration. On one hand, in the groups with high immune scores, the local immune signature may present a stronger immune phenotype. On the other hand, a weaker immune infiltration is seen in groups with low immune scores.

**Table 1:** Relative proportion of 22 types of immune cells in high and low immune score groups.

Immune cell type	Low immune score (%)	High immune score (%)	P values
B cells naive	2.78% ± 3.03%	2.24% ± 2.38%	0.483
B cells memory	0.13% ± 0.35%	0.50% ± 1.35%	0.461
Plasma cells	4.84% ± 5.98%	4.52% ± 5.97%	0.857
T cells CD8	4.98% ± 5.48%	9.27% ± 6.50%	<0.001
T cells CD4 naive	0.17% ± 1.19%	0.05% ± 0.64%	0.386
T cells CD4 memory resting	18.08% ± 7.02%	17.69% ± 6.24%	0.584
T cells CD4 memory activated	0.21% ± 0.97%	0.91% ± 2.14%	0.001
T cells follicular helper	3.69% ± 2.85%	3.06% ± 2.20%	0.240
T cells regulatory (Tregs)	4.97% ± 2.88%	5.53% ± 3.08%	0.326
T cells gamma delta	0.19% ± 1.06%	0.19% ± 0.66%	0.364
NK cells resting	0.45% ± 1.00%	0.56% ± 1.26%	0.907
NK cells activated	4.15% ± 2.97%	4.37% ± 3.33%	0.807
Monocytes	1.55% ± 2.10%	2.95% ± 4.14%	0.011
Macrophages M0	24.82% ± 15.08%	16.44% ± 11.96%	0.001
Macrophages M1	7.07% ± 4.15%	9.05% ± 4.20%	0.008
Macrophages M2	13.37% ± 6.09%	17.19% ± 6.86%	0.001
Dendritic cells resting	0.25% ± 1.05%	1.22% ± 2.27%	<0.001
Dendritic cells activated	4.80% ± 6.88%	1.24% ± 2.53%	0.003
Mast cells resting	1.66% ± 3.62%	1.42% ± 1.86%	0.056
Mast cells activated	1.47% ± 2.42%	1.01% ± 2.34%	0.164
Eosinophils	0.17% ± 0.50%	0.08% ± 0.39%	0.141
Neutrophils	0.20% ± 0.53%	0.50% ± 1.10%	0.001

### Identification of Immune-Related Differentially Expression Genes With Prognostic Value Between Tumor and Normal Tissues in OV

**Figure 2:**
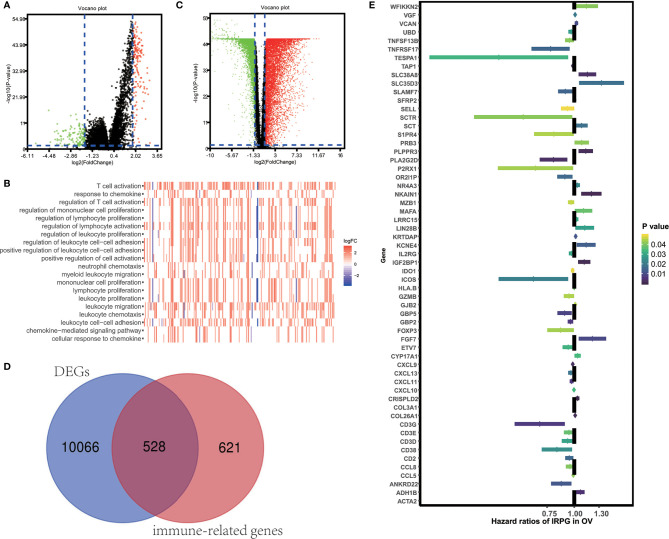
Identification of immune-related prognostic expressed genes in OV. **(A)** Genes with differential expression between the low and high immune score groups. **(B)** Top GO terms which DEGs between high and low immune score groups enriched. **(C)** Genes with differential expression between tumor and tumor adjacent tissues. **(D)** Among 10594 up-regulated or down-regulated in tumor tissue, 528 are immune-related genes. **(E)** The Hazard ratios of identifying immune-related prognostic genes.

To unveil the OV profiles’ association with immune scores, we executed a differential expression analysis occurring in both high and low immune score groups (|*log*_2_ *FC|* > 1 and FDR<0.05) using the “limma” package. Volcano plots (**Figure 2A**) reveal the unique gene expression profiles of patients under the low and high immune score groups. To outline the potential function of the DEGs, we performed functional enrichment analysis of the 1049 up-regulated or down-regulated genes in high immune score group. As expected, top gene ontology (GO) terms identified included T cell activation, leukocyte migration, leukocyte cell−cell adhesion, regulation of T cell activation, regulation of lymphocyte activation, regulation of leukocyte proliferation (**Figure 2B**), which suggest that these genes significantly relate to immune signal transduction. Thus, we specified differentially expression genes between high and low immune score groups as immune-related genes for further study. “Limma” package also helps us to recognize genes that are differentially expressed in tumor and normal tissues. As is shown in **Figure 2C**, 10594 differentially expression genes were obtained (|*log*_2_ *FC|* > 1 and FDR<0.05). Among the 10594 DEGs investigated, 528 are immune-related genes (**Figure 2D**). Then, we tried to gauge the prognostic predictive ability of these immune-related DEGs and performed our analysis using the univariate Cox regression, which discovered the significant association of 59 out of 528 immune-related DEGs to overall survival (**Figure 2E**). P value<0.05 was set as the cut-off value. **Table 2** exhibits genes that first report in OV.

**Table 2:** First reported immune microenvironment- related genes in OV.

Gene Symbol	logFC		FDR	HR	P value
ANKRD22		2.950	0.000	0.871	0.012
CD3D		2.314	0.000	0.933	0.029
CD3G		2.097	0.000	0.694	0.007
COL26A1		3.723	0.000	1.010	0.002
CRISPLD2		-2.519	0.000	1.036	0.000
ETV7		1.885	0.000	0.938	0.032
GBP5		1.516	0.000	0.903	0.009
GJB2		2.093	0.000	1.010	0.045
KCNE4		-1.765	0.000	1.134	0.014
KRTDAP		-3.876	0.000	1.015	0.012
MAFA		3.469	0.000	1.103	0.037
MZB1		2.032	0.000	0.967	0.046
NKAIN1		2.423	0.000	1.199	0.001
OR2I1P		6.265	0.000	0.906	0.016
PLA2G2D		3.725	0.000	0.803	0.003
PLPPR3		1.758	0.009	1.130	0.002
PRB3		3.729	0.000	1.082	0.042
S1PR4		1.786	0.000	0.806	0.045
SLAMF7		2.638	0.000	0.909	0.015
SLC35D3		3.600	0.000	1.334	0.017
SLC38A8		1.553	0.000	1.149	0.003
TESPA1		-2.817	0.000	0.450	0.033
TNFSF13B		2.562	0.000	0.951	0.041
WFIKKN2		-7.555	0.000	1.137	0.041

logFC, log fold change (tumor tissues vs. normal tissues). FDR, false discovery rate.

### Characteristics of 59 Immune-Related Prognostic Genes

**Figure 3:**
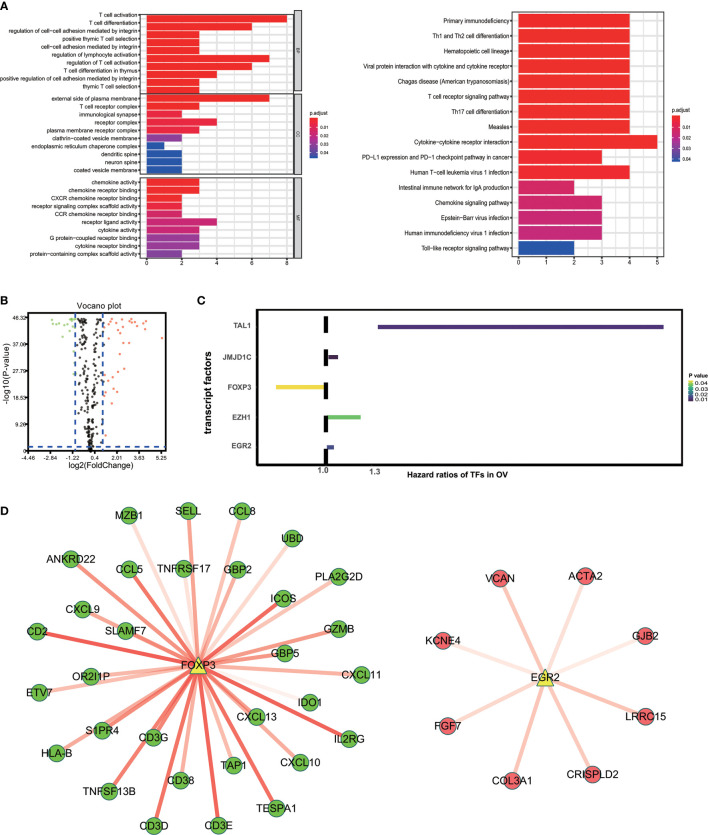
Characteristics of 59 immune-related prognostic genes. **(A)** Top GO terms and KEGG pathways enriched by IRPG. **(B)** Volcano plots reveal differentially expressed transcript factors (TFs) between tumor and adjacent tissues. **(C)** The Hazard ratios of identified TFs with prognostic value. **(D)** TF-based regulatory schematic.

“External side of plasma membrane”, “T cell activation” and “chemokine activity” were most frequently enriched in GO terms among cellular components, biological processes and molecular functions. Kyoto Encyclopedia of Genes and Genomes (KEGG) analysis were most significantly associated with cytokine-cytokine receptor interaction (**Figure 3A**). To study possible molecular mechanisms corresponding to the clinical significance of our prognostic immune-related genes, we assessed the regulatory mechanisms of these genes. **Figure 3B** shows our examination of 318 expression profiles of transcription factors (TFs). From this, we determined 95 differentially expressed genes between tissues from OV and normal ovary. Out of these 95 TFs, 5 were linked to the overall survival of patients with ovarian cancer tissue at p-value<0.05 (**Figure 3C**). Based on these 5 TFs and 59 prognostic genes that are immune-related, we created a regulatory network. We established the cut-off value to be at a correlation score of greater than 0.3 and P-value<0.001. As is shown in **Figure 3D**, the intense illustration by the TF-based regulatory schematic of the regulatory connections amongst these prognostic immune-related genes is evident. FOXP3 is a key TF that has a positive correlation with high-risk immune-related genes (HR>1). Meanwhile, EGR2 is significantly correlated with low risk immune-related genes (HR<1).

### Construction of Immune Prognostic Model

**Figure 4:**
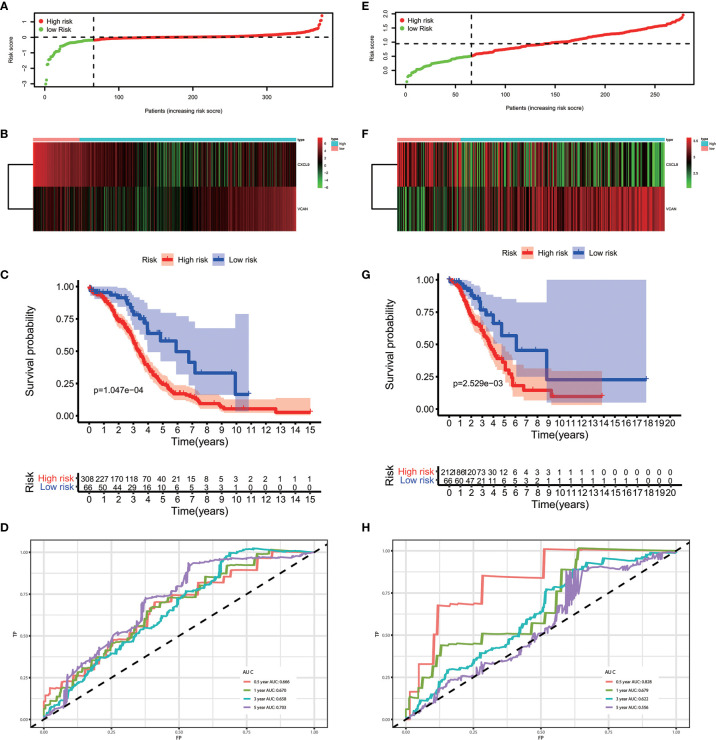
The performance of our IPM in TCGA and GEO cohort. **(A, E)** Rank of risk score and distribution of groups. **(B, F)** The expression of included genes, CXCL9 and VCAN. **(C, G)** Survival curve of risk score. Patients with a higher risk score had a higher overall survival than their counterparts. **(D, H)** The time independent ROC.

The results of the multivariate Cox regression analysis enabled us to establish a prognostic signature to classify the patients with ovarian cancer into two groups with discrete clinical outcomes depending on their risk score. The formula is: risk score= [Expression level of CXCL9 * (-0.01752)] + [Expression level of VCAN * (0.02584)]. We computed for each patient’s risk score and categorized them according to the level of risk according to the optimal cut off point provided by the X-tile software. 0.180 was served as the cutoff value to classify the OV patients into high and low risk groups. **Figures 4A, B** shows the risk score of the publicly available samples and the expression of included genes. Survival curve (**Figure 4C**) reveals that patients with a lower risk score had a higher overall survival than their counterparts (P<0.001). The time independent ROC (**Figure 4D**) shows a good performance of our immune prognostic model, the area under the ROC curve was 0.666 at 0.5 year, 0.670 at 1 year, 0.658 at 3 year and 0.703 at 5 year. In addition, the multivariate Cox analysis (**Table 3**) revealed that our IPM is an independent predictor for prognosis of OVs (HR=2.844, P<0.001).

**Table 3:** Associations with overall survival and clinicalopathologic characteristics in TCGA patients using COX regression.

Clinical characteristics	HR	95%Cl(low)	95%Cl(high)	P value
Age (continuous)	1.022	1.01	1.035	<0.001
Radiation therapy (Yes vs. No)	1.293	0.18	9.295	0.798
Pharmaceutical therapy (Yes vs. No)	0.544	0.314	0.941	0.03
Histological grade (High vs. Low)	1.214	0.817	1.803	0.338
Risk score (continuous)	2.82	1.773	4.486	<0.001
Multivariate analysis				
Age (continuous)	1.024	1.011	1.037	0.000
Pharmaceutical therapy (Yes vs. No)	0.421	0.242	0.735	0.002
Risk score (continuous)	2.844	1.810	4.468	0.000

### Validation of Performance of IPM in GEO Cohort

To evaluate whether the performance of IPM was robust, we downloaded another OV cohort (GSE8191) from GEO database as test cohort, which includes 285 OV patients. According to risk scores calculated by the same formula and the optimal cutoff point (0.510), patients in train cohort were grouped into high-risk and low-risk group. As is shown in **Figures 4E–H**, people assigned into low-risk group had an obviously favorable prognosis than which assigned into high-risk group (P=0.003). Meanwhile, the AUC of IPM at 0.5, 1, 3, and 5 years was 0.722, 0.679, 0.622, and 0.556 respectively. In multivariate Cox analysis (**Table 4**), IPM is also an independent predictor for prognosis of OVs (HR=1.774, P=0.006), along with Figo stage (HR=1.748, P=0.006) and age (HR=1.027, P=0.007).

**Table 4:** Associations with overall survival and clinicalopathologic characteristics in GEO patients using COX regression.

Clinal characteristics	HR	95%Cl(low)	95%Cl(high)	P value
Histological subtype (serous vs. others)	0.115	0.016	0.823	0.031
Age (continuous)	1.026	1.006	1.046	0.011
Figo stage (III&IV vs. I&II)	2.144	1.487	3.093	0.000
Histological grade (High vs. Low)	1.337	0.975	1.835	0.072
Platin treatment (Yes vs. No)	3.204	1.015	10.118	0.047
Taxol treatment (Yes vs. No)	0.737	0.492	1.103	0.138
Neoadjuvant treatment (Yes vs. No)	1.495	0.726	3.079	0.275
Risk score (continuous)	1.679	1.129	2.497	0.011
Multivariate Cox analysis				
Histological subtype (serous vs. others)	0.172	0.024	1.246	0.081
Age (continuous)	1.027	1.007	1.048	0.007
Figo stage (III&IV vs. I&II)	1.748	1.176	2.596	0.006
Platin treatment (Yes vs. No)	2.755	0.856	8.866	0.089
Risk score (continuous)	1.774	1.176	2.677	0.006

### Immune Landscape Between the Low- and High-Risk OV Patients

**Figure 5:**
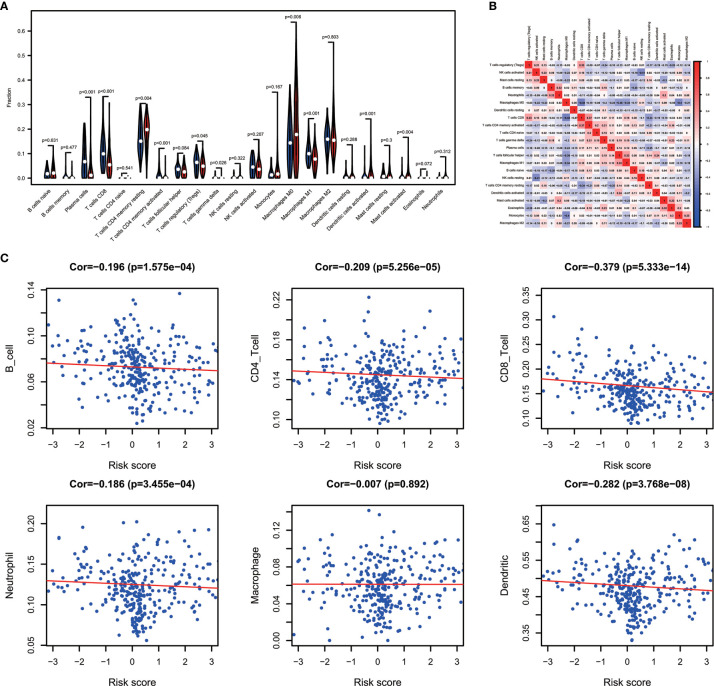
Immune landscapes of the low- and high-risk OV patients. **(A)** The proportion of different types of immune cells in high and low risk groups. Low-risk groups share a higher fraction of Plasma cells (P<0.001), T cells CD8 (P<0.001), T cells CD4 memory activated (P<0.001), T cells regulatory (P=0.045), T cells gamma delta (P=0.026) and Macrophages M1 (P<0.01). **(B)** Correlation matrix of all 22 immune cell proportions. **(C)** Correlations between risk score and immune infiltrating level of 6 types of immune cells.

Combining the CIBERSORT methodology with gene expression profiling acquired from TCGA database, we evaluated the variations among low and high-risk OV patients in terms of the immune infiltration of 22 different kinds of immune cells. **Figure 5A** shows the results of immune landscape obtained from 202 OV patients after filtering (20). Within and between groups, the proportion of immune cells in OV varies (**Figure 5A**, **Table 5**). Low risk groups share a higher fraction of Plasma cells (P<0.001), T cells CD8 (P<0.001), T cells CD4 memory activated (P<0.001), T cells regulatory (P=0.045), T cells gamma delta (P=0.026) and Macrophages M1 (P<0.01). The proportions of different types of immune infiltrating cells were weakly to moderately correlated (**Figure 5B**). In addition, the outcome of correlation analysis between risk score and the abundance of immune infiltrating cells was exhibited in **Figure 5C**. The density of B cells, CD4+ T cells, CD8+ T cells, neutrophil cells, dendritic cells in OV were significantly associated with risk score. Thus, the results suggest that our IPM could serve as a predictor of the level of immune infiltration. Also, the heterogeneity and abnormality of immune infiltration amongst OV patients are possible prognostic indicators and targets for immunotherapy, which may also have vital clinical relevance.

**Table 5:** Relative proportion of 22 types of immune cells in high and low risk score groups.

Immune cell type	Low risk group (%)	High risk group (%)	P values
B cells naive	2.05% ± 1.90%	2.55% ± 2.72%	0.631
B cells memory	0.48% ± 1.54%	0.42% ± 1.10%	0.477
Plasma cells	7.57% ± 6.50%	3.40% ± 5.41%	<0.001
T cells CD8	11.48% ± 6.99%	6.81% ± 5.76%	<0.001
T cells CD4 naive	0.03% ± 0.25%	0.01% ± 0.08%	0.541
T cells CD4 memory resting	16.07% ± 6.08%	18.62% ± 5.96%	0.004
T cells CD4 memory activated	1.48% ± 2.59%	0.42% ± 1.41%	<0.001
T cells follicular helper	3.38% ± 2.10%	2.88% ± 2.26%	0.084
T cells regulatory (Tregs)	6.02% ± 3.36%	5.06% ± 2.83%	0.045
T cells gamma delta	0.31% ± 0.75%	0.08% ± 0.42%	0.026
NK cells resting	0.38% ± 0.83%	0.64% ± 1.35%	0.322
NK cells activated	4.68% ± 3.38%	4.07% ± 3.30%	0.207
Monocytes	1.92% ± 2.14%	2.96% ± 4.27%	0.167
Macrophages M0	14.22% ± 8.89%	20.63% ± 14.26%	0.006
Macrophages M1	10.37% ± 4.11%	7.81% ± 4.14%	<0.001
Macrophages M2	15.76% ± 5.33%	16.45% ± 7.39%	0.803
Dendritic cells resting Dendritic cells activated Mast cells resting	0.84% ± 1.58%	1.03% ± 2.29%	0.288
	0.68% ± 2.14% 1.36% ± 1.67%	2.64% ± 4.75% 1.42% ± 2.36%	0.001 0.300
Mast cells activated	0.54% ± 1.48%	1.48% ± 2.84%	0.004
Eosinophils	0.11% ± 0.56%	0.10% ± 0.36%	0.072
Neutrophils	0.26% ± 0.50%	0.51% ± 1.14%	0.312

**Figure 6:**
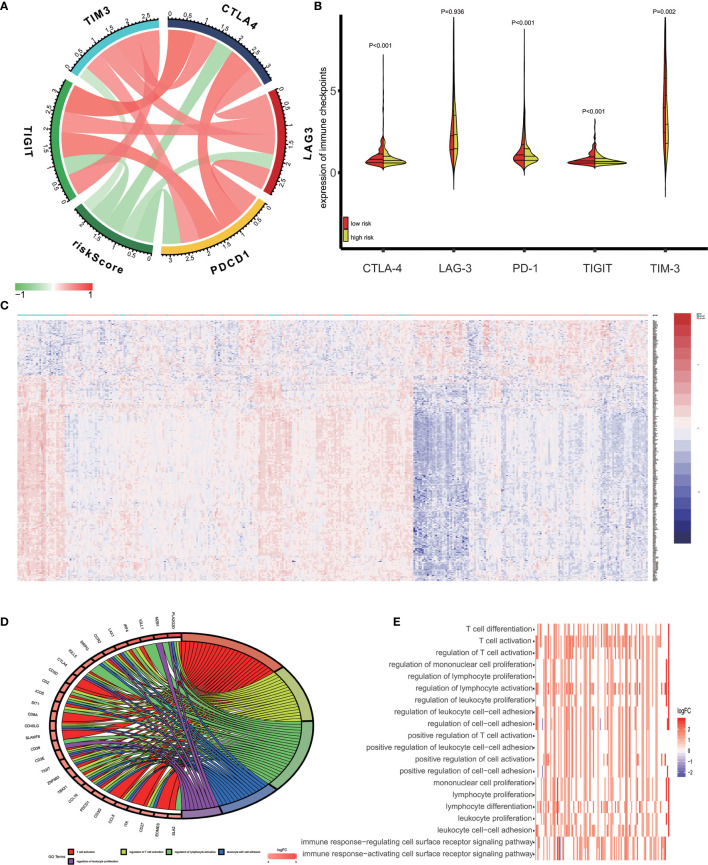
Enrichment analysis of the immune prognostic model. **(A)** Correlation between expression of several prominent immune checkpoints and risk scores. **(B)** Comparison of the expression of selected immune checkpoint in high and low risk groups. **(C)** Heatmap of differentially expressed genes in high and low risk groups. **(D)** Circular plot of the biological processes enriched for the immune genes. **(E)** GO enrichment analysis.

Recent studies reported that tumor cells acquire escape mechanisms to evade host immunity in the tumor microenvironment. The immune checkpoints play a significant role to promote tumor enhancement by tumor immunosuppressive effects (28). Some prominent immune checkpoints could serve as a biomarker for predicting the efficacy of immunotherapy (29). Therefore, we calculated the Pearson’s correlation between expression of several prominent immune checkpoints (CTLA−4, LAG−3, PD−1, TIGIT and TIM-3) and risk score (**Figure 6A**). The results showed that patients’ risk score was significantly negative correlated to expression of immune checkpoints (P<0.05) and the expression of immune checkpoints are positively correlated between themselves. In addition, we access the expression of CTLA-4, LAG-3, PD-1, TIGIT and TIM-3 in high-risk and low-risk groups. As is shown in **Figure 6B**, the expression of CTLA-4, PD-1, TIGIT and TIM-3 in low-risk groups was significantly higher than in high-risk groups.

### Different Pathways Enriched in High-Risk and Low-Risk Groups

In this study, we used the GO analysis to study the biological impacts of the IPM. For the low and high-risk groups of OV patients, 340 genes were differentially expressed (**Figure 6C**) (|*log*_2_ *FC|* > 1 and FDR<0.05). These genes were determined to be associated with the risk scores and underwent the GO analysis for the specification of their possible biological implications (FDR< 0.0001). The results revealed the underlying mechanism of the genes associated with risk score, which primarily play a role in lymphocyte differentiation, activation and proliferation of the immune system. The pathway enriched included T cell differentiation, T cell activation, regulation of T cell activation, regulation of mononuclear cell proliferation, and regulation of lymphocyte proliferation (**Figures 6D, E**).

## Discussion

We acknowledge that in the context of immunotherapies, research on the importance of immune-related biomarkers and clinical prognosis has become increasingly prevalent (30, 31). Previous studies revealed that recurrent ovarian cancer patients could have some significant immune subset of patients really suitable to have immune therapeutic option when secondary cytoreduction is impossible (32, 33). However, specific investigations on the whole profiling of related genomes that delve into tumor-infiltrating immune cells related genes that are relevant to ovarian cancer have yet to be executed. This extensive and cohesive analysis of immune-related genes in ovarian cancer improves our appreciation for their clinical relevance while it highlights possible molecular mechanisms.

Many previous study have applied ESTIMATE algorithm to various types of cancer, including breast cancer (11), colon cancer (12), gastric cancer (13) and brain cancer (14). However, the utility of OV has not been studied in detail. In this study, to validate its performance in OV, we estimated the difference of immune infiltration between high and low immune score groups and discovered that the proportion of subtypes of immune cells is obviously varied. In correlation analysis based on TIMER reanalyzes gene expression data, immune score moderately to strongly correlates to immune infiltration level. Although the prognostic value is not significant, immune score calculated by ESTIMATE algorithm is still a reliable predict factor of immune infiltration level and significantly relates to the subtypes of immune microenvironment cells.

Then by the comparison the gene expression data of tumor vs. normal tissues and high vs. low immune score tissues, we extracted a list of immune-related genes and demonstrated that they were significantly concerned in human immune response and regulation of lymphocytes, as shown in enrichment analysis of GO terms. Then, we identified genes with prognostic value among them using univariate Cox analysis. Importantly, a few of these genes were first reported in OV, which could serve as potential biomarkers for OV patients and provide a new landscape for immunotherapy. To discover the fundamental mechanisms at the molecular level that corresponds to the possible clinical significance, we established a network mediated by TF to identify vital TFs that could regulate genes we identified as immune-related prognostic genes. In this network, FOXP3 and EGR2 were notably acknowledged. Previous immunological researches have revealed that FOXP3 and EGR2 serve as transcript factors that play important roles in regulation of lymphocyte function. Study of breast cancer suggested that FOXP3 function as a key tumor suppressor through the up-regulation of CXCR4 and down-regulation of CXCL12, which thereby stimulate cell migration (34). Furthermore, FOXP3, is acknowledged as a major and specific marker of Tregs, the cellular expression of which is correlated with suppressive activities. EGR2 is highly correlated members of the Egr zinc finger transcription factor family with significant function in regulating the self-tolerance of lymphocytes and the differentiation of T cells and NKT cells (35–37). Above all, the coexpression and differential expression based regulatory networks of transcript factors and immune-related prognostic genes we constructed may provide a great help to direct future mechanism analysis.

Considering that the immune score could not significantly predict the clinical outcome, thus we focused on constructing a prognostic signature based on these immune-related prognostic genes and produced an IPM that is 2-gene-based, which could assess OV patients that are at a high risk of developing poor prognoses in the future. In fact, CXCL9 and VCAN, which constitute our IPM has been described as the promising therapy target. CXCL9 is an IFN-γ-inducible chemokine as well as one of the main ligands for CXCR3 (38). The increasing expression level of CXCR3 could accelerate the accumulation of tumor microenvironment (TEM) cells by helping TEM cells rapidly migrating into inflamed tissues (39). Another previous study revealed a close association between the CXCL9 and CCL5 expressions in OV and other cancers. Their coexpression, which had a phenotype that was molecularly immunoreactive, is correlated with the TEM cells (40). VCAN is an enormous matrix comprised of proteoglycan with activities classified as immunoregulatory. It amasses in the tumors’ extracellular matrix (41). Also, it is known for its contribution toward inflammations that are either cancerous or non-cancerous through its stimulation of inflammatory mediators derived by leukocytes (42, 43). Moreover, it influences immunodeficiency through the dysfunction of dendritic cell (DC) (44). The infiltration of the T-cell is promoted by the versikine and matrikine that were derived from VCAN. The process involved the regulation of a unique DC subset, known as the Batf3-dependent dendritic cells, which is vital for the migrating of effector T cell (45), reaction to numerous modes of immunotherapy (46–48), and antitumor immunity mediated by the T cell (49, 50). Additionally, the literature on colorectal cancer and multiple myeloma recommends the antagonistic feature of versikine towards the tolerogenic actions of the whole VCAN. Hence, this could generate a promising antitumor strategy (51, 52).

The high density of tumor-infiltrating immune cells is associated with positive clinical prognosis and enhanced response rates to checkpoint inhibitor therapy, which is a main form of presentation immunotherapy for cancer (53). Here, we evaluated the proportion of different types of TIICs, immune infiltrating level and the expression of immune checkpoint in low- and high-risk group to identify the difference of immune mechanisms between low- and high-risk scores and possible use of our IPM to immunotherapy in ovarian cancer. The results indicated that low risk score OVs contained a higher fraction of T cells regulatory (Tregs), T cells CD8, Macrophages M1, T cells CD4 memory activated than high risk score patients. The immune score is negatively correlated to the infiltration level of B cell, CD4+ T cell, CD8+ T cell, neutrophil and Dendritic. Interestingly, the expression of several prominent immune checkpoints (CTLA-4, PD-1, TIGIT, TIM-3) is also higher in low risk score groups. CD8+ T cell is a main kind of effector cell in antitumor immune response, the important role of which in suppressing tumor has been publicly recognized (54). Tregs are involved in cancers and many other autoimmune diseases and also be known as the immunosuppressive subset of CD4+ T cells that maintain the immune homeostasis by suppressing the function of T cells (55). The various types of effector lymphocytes are suppressed by Tregs migrating into the inflammatory site (56). Thus, Tregs also expresses a function similar to immune checkpoints. The outcome suggested that low risk score patients suffer a stronger immunosuppress, although with a higher overall survival and a higher level of immune infiltration. Thus, in our IPM, the risk score was consistency with the antitumor ability of TIICs, revealing that the favorable prognosis of the low-risk patients may be caused by the higher proportion of immune effecter cells and a higher level of immune infiltration than high-risk patients. The expression of immune checkpoint is one of the most effective predictors of response rates to immunotherapy (57). Thus, compared with high-risk patients, these outcomes also indicate the increased benefits of the checkpoint inhibitor therapy toward low-risk patients, thereby further improving overall clinical outcomes for OV patients.

The GO terms enrichment analysis of differentially expressed genes between high and low risk groups revealed the difference of local immune signature between these two groups. 29 Genes most up-regulated in low risk groups significantly enriched in 5 immune-related pathways, including T cell activation, regulation of T cell activation, regulation of lymphocyte activation, leukocyte cell-cell adhesion and regulation of leukocyte proliferation. Additionally, the expression of CTLA-4, TIGIT and PDCD1 is significantly higher in low risk groups, suggesting that patients in the low risk group suffering a stronger immunosuppress. These results are consistent with our previous discovery, namely low risk score is associated with higher immune infiltration level and intense immunosuppress.

Our study provides new insights into the OV immune microenvironment by mining a list of novel immune microenvironment-associated genes. Furthermore, we develop a straightforward 2 gene-based immune-related prognostic models that reflect the overall immune landscape and have independent prognostic significance for OV patients. However, our study has some limitation. First, transcriptional changes are also the main contributors to functional alterations (58). When the studies are founded on genomic alterations, these are not representative of the overall situation. Second, as samples from TCGA (n=388) and GEO (n=285) of our study were relatively small, larger sample size data are needed for verification. Additionally, our retrospective study produced results that need to be further verified by prospective studies.

## Data Availability Statement

The datasets presented in this study can be found in online repositories. The names of the repository/repositories and accession number(s) can be found in the article/supplementary material.

## Author Contributions

WW and QW designed the experiments and wrote the paper. ZW and YX analyzed the gene expression data. SR and HS interpreted the figures. YX and WS inspected the work. All authors contributed to the article and approved the submitted version.

## Funding

This study was funded by the National Natural Science Foundation of China (No.81802606).

## Conflict of Interest

The authors declare that the research was conducted in the absence of any commercial or financial relationships that could be construed as a potential conflict of interest.
